# Functional Metal Organic Framework/SiO_2_ Nanocomposites: From Versatile Synthesis to Advanced Applications

**DOI:** 10.3390/polym11111823

**Published:** 2019-11-06

**Authors:** Mengyu Ma, Liangyu Lu, Hongwei Li, Yuzhu Xiong, Fuping Dong

**Affiliations:** Department of Polymer Materials and Engineering, Guizhou University, Guiyang 550025, China; mmymamengyu@163.com (M.M.); gs.lylu17@gzu.edu.cn (L.L.); lhwnoonoo123@163.com (H.L.); yzxiong@gzu.edu.cn (Y.X.)

**Keywords:** metal organic frameworks, silica, chromatographic column separation, gas adsorption, catalysis, biomedicine

## Abstract

Metal organic frameworks (MOFs), also called porous coordination polymers, have attracted extensive attention as molecular-level organic-inorganic hybrid supramolecular solid materials bridged by metal ions/clusters and organic ligands. Given their advantages, such as their high specific surface area, high porosity, and open active metal sites, MOFs offer great potential for gas storage, adsorption, catalysis, pollute removal, and biomedicine. However, the relatively weak stability and poor mechanical property of most MOFs have limited the practical application of such materials. Recently, the combination of MOFs with inorganic materials has been found to provide a possible strategy to solve such limitations. Silica, which has excellent chemical stability and mechanical properties, shows great advantages in compounding with MOFs to improve their properties and performance. It not only provides structured support for MOF materials but also improves the stability of materials through hydrophobic interaction or covalent bonding. This review summarizes the fabrication strategy, structural characteristics, and applications of MOF/silica composites, focusing on their application in chromatographic column separation, catalysis, biomedicine, and adsorption. The challenges of the application of MOF/SiO_2_ composites are addressed, and future developments are prospected.

## 1. Introduction

Metal organic frameworks (MOFs), also called porous coordination polymers (PCPs), as organic-inorganic hybrid materials with a 3D periodic grid structure formed by coordination bonds between metal ions or clusters and organic ligands, have developed rapidly in recent decades [1,2,3,4,5,6]. The structures of the originally prepared MOFs are not stable, and their skeletons easily collapse, limiting further research and application. In the 1990s, Yaghi et al. synthesized MOF-5, which retained the skeleton integrity after removing the guest molecules in the channel [7,8]. The successful synthesis of MOF-5 became a milestone in the development of MOFs. MOFs have the advantages of ultrahigh specific surface area, high and adjustable porosity, diverse structural composition, open metal sites, and chemical modifiability, which make them widely concerned in the fields of gas storage and separation, catalysis, energy, and drug-sustained release [9,10,11,12,13,14,15,16,17,18,19]. However, weak coordination bond composition results in the poor stability and low mechanical strength of many MOF materials, which greatly limit their application [20,21]. MOFs with higher stability can be obtained by synthesizing metal–nitrogen-containing MOFs with high bonding energy, by synthesizing MOFs with metals with high coordination numbers, or by post-synthetic modification using organic linkers [22,23,24]. Currently, in practical applications, especially in high-temperature and high-humidity industrial environments, enhancing the thermal stability, water stability, chemical stability, and mechanical strength of MOFs while maintaining high specific surface area and high activity remains a big challenge [23,25,26]. To solve this problem, researchers have attempted to compound MOFs with other materials to form MOF nanocomposites [27,28,29,30,31,32,33]. MOF composites are two-phase or multiphase composites based on MOFs as matrixes with polymers, metals, silica, graphene, or carbon nanotubes with different properties as the reinforcement phase [34,35,36,37,38,39,40,41,42,43,44]. Among them, silica, especially porous silica, is very suitable for the preparation of composite materials to improve the performance of MOFs due to its high stability and structural adjustability [45,46,47,48]. Silica not only provides structured support for MOFs but also improves the stability of materials through hydrophobic interaction or covalent bonding. The compound strategy of MOF/SiO_2_ composites generally includes an in situ method, where MOF crystals are grown on the prepared silica, and the sol–gel method, where a silica layer is coated on the synthesized MOF crystals [49].

MOF/silica composites demonstrate excellent performance in various applications. Especially, as the stationary phase of the chromatographic column, the highly uniform core–shell-structured MOF/silica composite could improve the uneven packing of MOF particles and greatly enhance the separation efficiency [50]. Silica has a guiding effect on the growth of MOF crystals and could effectively design and control the morphology of MOFs. The interaction between silanol groups on the silica precursor and metal center can cause structural changes in the material and lead to the increase in surface area and micropore volume, which will facilitate gas adsorption applications [51]. In addition, MOF/silica composites containing different open metal sites can not only act as catalysts but also support noble metal particles, which have great potential in the field of catalysis [52]. MOF/silica composites have also been utilized for drug delivery, angiography, diagnosis, treatment, and other biomedical fields [53]. Especially in the field of next-generation drug-sustained release, MOF materials have great advantages due to their organic-inorganic hybrid structure, molecular horizontal structure, designability/adjustable porosity, and easy chemical modification. After compounding with silica, the biological stability and structural diversity of MOF materials can be improved and become more suitable for biomedical applications [54]. As shown in Table 1, the synthesis strategy and the advanced applications of MOF/SiO_2_ nanocomposites have been summarized in detail. Even though people have reviewed some composite materials containing MOFs for some special applications [55,56,57,58,59], the review of the specific topic of MOF/SiO_2_ composite materials still seems necessary, due to the rapid development in this field. Herein, the fabrication and properties of MOF/silica composites reported in recent years are summarized. Especially, their applications in the fields of chromatographic column separation, catalysis, adsorption, and biomedicine, have been analyzed carefully. The problems and challenges in the application of MOF/silica composites are pointed out.

## 2. General Strategies for MOF/Silica Composite

Silica, which has excellent chemical stability and mechanical properties, shows great advantages in the compounding with MOFs to improve their properties and performance. It not only provides structured support for MOF materials but also improves the stability of materials through hydrophobic interaction and covalent bonding [60]. The structure, morphology, surface chemistry, and thermal stability of the MOF/silica composite could be characterized by the techniques of X-ray diffraction (XRD), scanning electron microscope (SEM), transmission electron Microscopy (TEM), Fourier transform infrared spectroscopy (FTIR), thermal gravimetric analysis (TGA), etc. To fabricate MOF/SiO_2_ composites, three processes including in situ synthesis, sol–gel methods, and impregnation are usually utilized according to their application requirements.

**Figure 1:**
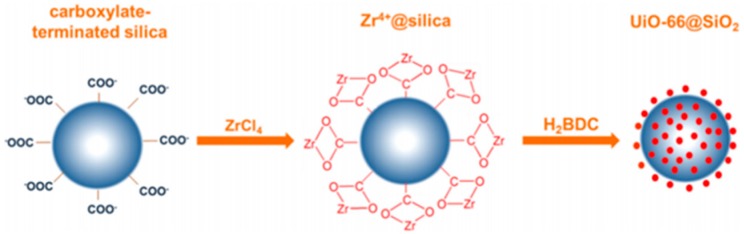
Synthetic procedure of UiO-66@SiO_2_ shell-core composites via in situ coating process. Reproduced from [61], with permission from Elsevier, 2019.

For the in situ process of coating MOF layers on silica particles, silica seeds are prepared first, and then MOF are grown on them by the one-pot or layer-by-layer assembly method. As shown in Figure 1, Gao et al. first prepared carboxylate-terminated silica, which adsorbed the Zr^4+^ on the silica surface and finally reacted with 1,4-benzenedicarboxylic acid (H_2_BDC) to form UiO-66@SiO_2_ [61]. In this process, uniform-sized silica worked as a support and offered MOFs regular shape and narrow size distribution, which are very important for versatile applications.

**Figure 2:**
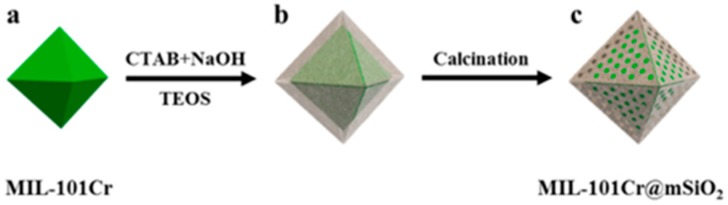
Schematic representation of the synthesis of MIL-101(Cr)@mSiO_2_ sample via a sol–gel process to grow silica layer on MOF crystal. (**a**) MIL-101Cr; (**b**) as-synthesized MIL-101(Cr)@mSiO_2_; (**c**) final MIL-101(Cr)@mSiO_2_. Reproduced from [80], with permission from American Chemical Society, 2018.

In the sol-gel method, MOF crystals are first synthesized, and then the silica precursors are hydrolyzed, condensed, and gelled to form porous SiO_2_ shells in the presence of MOFs [79]. The silica coating prepared using this strategy not only maintains the intrinsic good performance of the MOFs but also improves the stability and mechanical properties of the MOF materials. As Figure 2 shows, a very thin shell of hydrophobic mesoporous silica was encapsulated on the prepared MIL-101(Cr) crystals by sol–gel and calcination processes [80]. The MIL-101(Cr)@SiO_2_ was utilized to catalyze the oxidation reaction of indene with H_2_O_2_ in acetonitrile, and the activity of MIL-101(Cr)@SiO_2_ was found obviously higher than that of the pure MIL-101(Cr) sample. The turnover frequency (TOF) value of the MIL-101(Cr)@SiO_2_ (95.2 mmol g^−1^ h^−1^) is 1.24 times higher than that of the MIL-101(Cr) (76.8 mmol g^−1^ h^−1^). Moreover, after 1 h, the conversion of indene with the MIL-101(Cr)@SiO_2_ sample is 95%, while the conversion with the MIL-101(Cr) is only 77%. The porous silica shell facilitates the diffusion of reactants, which will improve the catalytic performance and catalytic stability of the MOFs.

**Figure 3:**
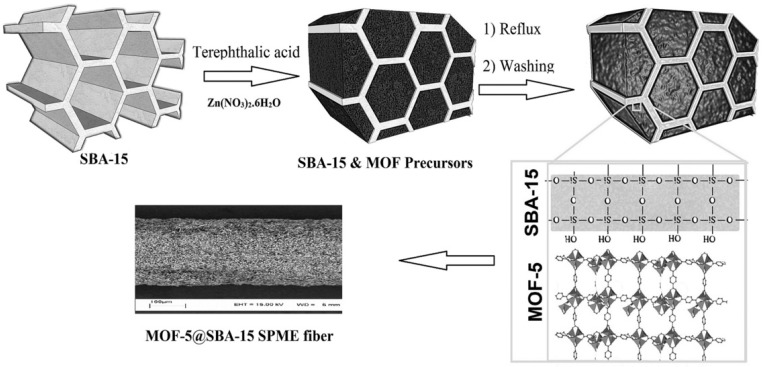
Synthesis of MOF-5@SBA-15 hybrid nanomaterial via an impregnation approach to grow MOFs only in the pores of porous silica materials. Reproduced from [83], with permission from Wiley-VCH, 2018.

An impregnation approach could be utilized to grow MOFs confined in the pores of porous silica materials to well control the morphology of MOF/silica composites [81,82]. Abolghasemi et al. first fabricated mesoporous silica (SBA-15) with uniform large pores and then impregnated the silica into the MOF precursors with suitable solvent and grew MOFs only on the pore surfaces (Figure 3) [83]. The obtained MOF-5@SBA-15 nanocomposite shows an enhanced sorbent capacity of small molecules originating from the properties of the nanocomposites, including its porous structure, large surface area, and homogeneous morphology. 

## 3. Application of MOF/Silica Composite

### 3.1. Application in Chromatographic Column Separation

Given the large specific surface area, adjustable pore structure, and large number of active metal sites, MOFs have great potential in separation applications, especially in chromatographic column separation [84,85,86,87,88]. However, the irregular shape and wide size distribution of MOF particles lead to the difficulty in column packing, low column efficiency, or high column pressure. Combining traditionally well-filled silica (which has excellent column-filling properties) with MOFs (which have excellent separation properties) is an excellent strategy to fabricate composites for the stationary phase of high-performance liquid chromatography (HPLC) [89,90,91,92]. The structure of the composite could greatly influence the separation ability of the materials. Exploring and optimizing the structure of MOF/SiO_2_ composites are important to improve the separation performance. Usually, MOF/SiO_2_ composites with a different structure for chromatographic column separation could be fabricated by impregnation, sol–gel or solvothermal process.

#### 3.1.1. MOFs Grown on the Pores of Porous SiO_2_ Particles

**Figure 4:**
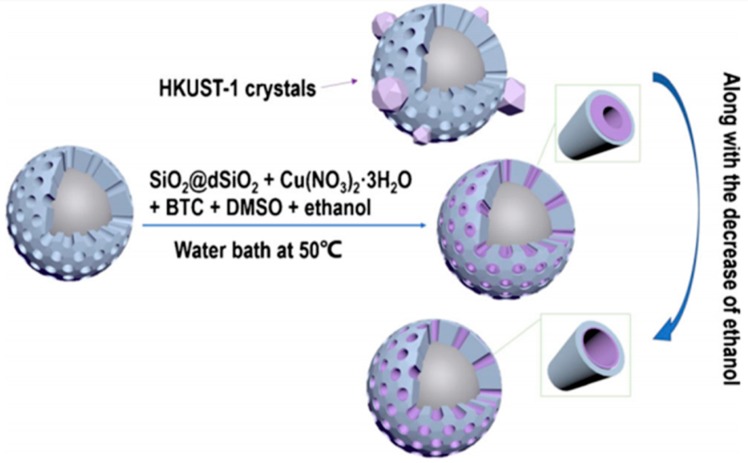
A schematic pathway for the modification of core-shell silica particles with HKUST-1 crystals. Reproduced from from [94], with permission from Springer, 2014.

To fabricate MOF/SiO_2_ composites for HPLC separation, porous silica particles are impregnated into the dispersion solution of MOF precursors and loaded with MOF nanocrystals in the pores after reaction to form the MOF/SiO_2_ composite. Ameloot et al. immersed porous silica beads in the precursor solution of HKUST-1, and after solvent evaporation, HKUST-1 crystals were grown in the pores of silica beads [93]. The obtained monodispersed MOF/SiO_2_ composite microspheres with a diameter of 3 µm showed high HPLC separation performance. Qu et al. also utilized carboxyl-modified mesoporous silica spheres as cores to grow a layer of HKUST-1 or ZIF-8 nanoscale films in the pores [62,94]. By adjusting the reaction condition, such as the volume of ethanol in the solvents, the formation of HKUST-1 crystals could be controllably coated only on the porous surface, and the thickness of the films could be controlled by changing the formation rate of nanocrystals (Figure 4). With the HKUST-1/SiO_2_ composite as a filler for the column of HPLC, a high separation efficiency as high as almost 140,000 plates per meter was achieved for the model analyte styrene.

#### 3.1.2. MOF Film on SiO_2_ Particles

**Figure 5:**
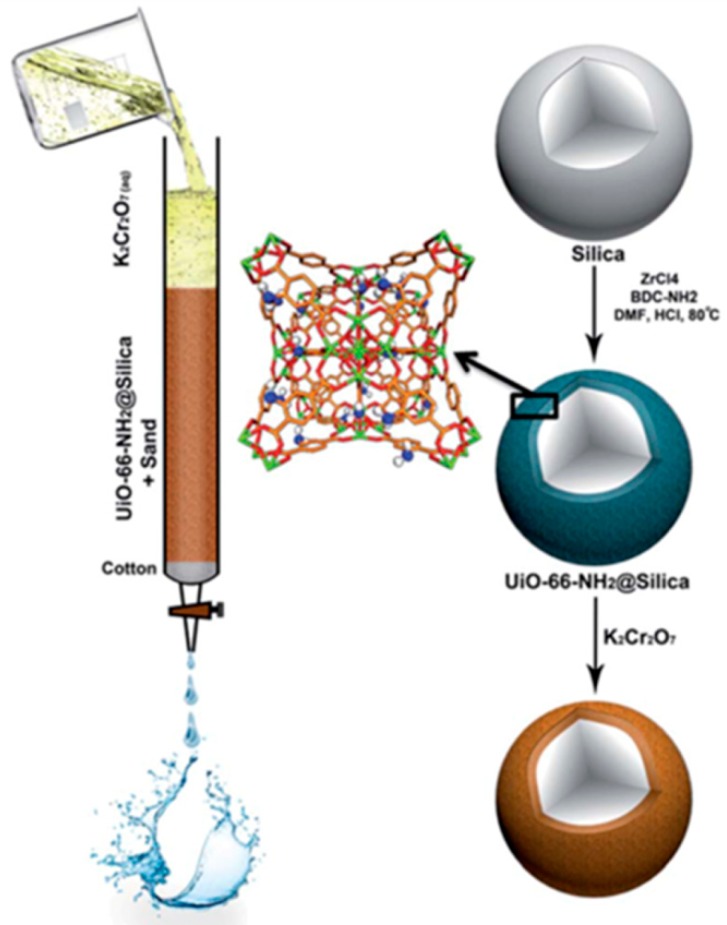
Hierarchical buildup of the UiO-66-NH_2_@silica and the column packing for (Cr_2_O_7_)^2−^ removal. Reproduced from [63], with permission from the Royal Society of Chemistry, 2018.

To increase the packing efficiency and sorbent–solute contact time, a suitable approach is to coat MOFs on silica, which is cheap, has a high surface area, and functions as a support. Through a one-pot synthesis process, El-mehalmey et al. wrapped a layer of amino-modified MOF materials on the surface of silica particles (Figure 5) to prepare a UiO-66-NH_2_@silica composite material. The composite was used as a porous solid phase of ion exchange column to improve the filling efficiency of the column and increase the contact time of the adsorbent and solute [63]. The results showed that the composite has an excellent uptake capacity of Cr(VI) ions in the chromatographic column. Even under the interference of competing anions, such as chloride, bromide, nitrate, and sulfate, the ion exchange column could effectively eliminate Cr(VI) ions. Yan et al. prepared mesoporous composite materials of MOF crystals on silica particles with the aid of surfactant cetyltrimethylammonium bromide (CTAB) by the solvothermal method and utilized them as new stationary phases for liquid chromatography (LC) [64,95]. The results showed that the isomers could be separated quickly and efficiently. Tanaka et al. prepared chiral (R)-MOF–silica composites using chiral organic ligands and copper nitrate or zinc nitrate as raw materials in the presence of monodispersed silica spheres by a one-step solvothermal method [96,97]. As a stationary phase of enantiomers in HPLC, this chiral composite material has excellent selectivity and high separation efficiency for various chiral sulfoxides. In addition, using the sol–gel method and supercritical CO_2_ drying method, Nuzhdin et al. prepared an HKUST-1@SiO_2_ aerogel composite material and used it in the stationary phase of conventional LC to efficiently separate unsaturated hydrocarbon from saturated aliphatic hydrocarbon [98].

#### 3.1.3. MOF Particles on SiO_2_ Particles

**Figure 6:**
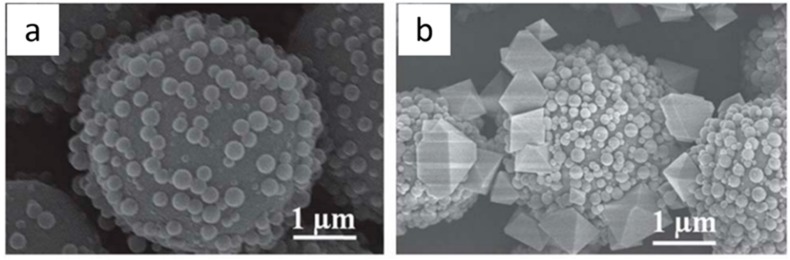
SEM images of (**a**) as-prepared spheres-on-sphere (SOS) silica particles particles and (**b**) HKUST-1@SOS-SiO_2_ particles. Reproduced from [103], with permission from the Royal Society of Chemistry, 2013.

Recently, superficially porous particles with a solid core and porous shell as the stationary phase for separation columns exhibited higher separation performance compared with only solid or porous materials [99,100,101,102]. Ahmed et al. utilized silica microspheres modified with amino or carboxyl groups as the template core to synthesize copper-based HKUST-1 nanocrystals on them and finally obtained HKUST-1/SiO_2_ microspheres with regular morphology [103]. As the stationary phase for fast and HPLC (Figure 6), the composite materials combined the filling and supporting function of the silica with the large number of open active metal sites of MOFs, achieving a high-efficiency separation of HPLC, which can rapidly separate toluene/ethylbenzene/styrene in 1.5 min. The same group also prepared a ZIF-8/SiO_2_ composite with stable ZIF-8 as the shell, which demonstrated high column separation efficiency of the aromatic mixture up to 19,000 plates/m due to the large specific surface area and its π–π interaction with the aromatic compound [104].

The MOF/silica composite mainly combines the advantages of silica microspheres (stability and suitable morphology) and MOFs (large specific surface area, adjustable pore structure, modifiability, and a large number of active metal sites), indicating its high application potential for chromatographic column separation. To improve the separation performance, exploring and optimizing the structure of MOFs/SiO_2_ composites are important. 

### 3.2. Application in Gas Adsorption

MOF materials have been widely used in the field of gas adsorption due to their high specific surface area, adjustable pore size, and pore surface, especially in the adsorption of carbon, sulfur, and nitrogen-containing gases [105,106,107]. The silica in the MOF/silica composites acts as a structure-directing agent to guide the growth of MOFs into a regular morphology and provides protection for MOFs to improve their stability [65]. The composite of porous/mesoporous silica and MOFs is expected to further reduce the mass transfer resistance of target molecules, such as gases, and accelerate the diffusion of target molecules, such as carbon dioxide. The synergistic effect between silicon dioxide and MOFs can also enhance the interaction between materials and gas molecules, thereby improving the gas adsorption efficiency of materials [108].

#### 3.2.1. Carbon Dioxide Adsorption

**Figure 7:**
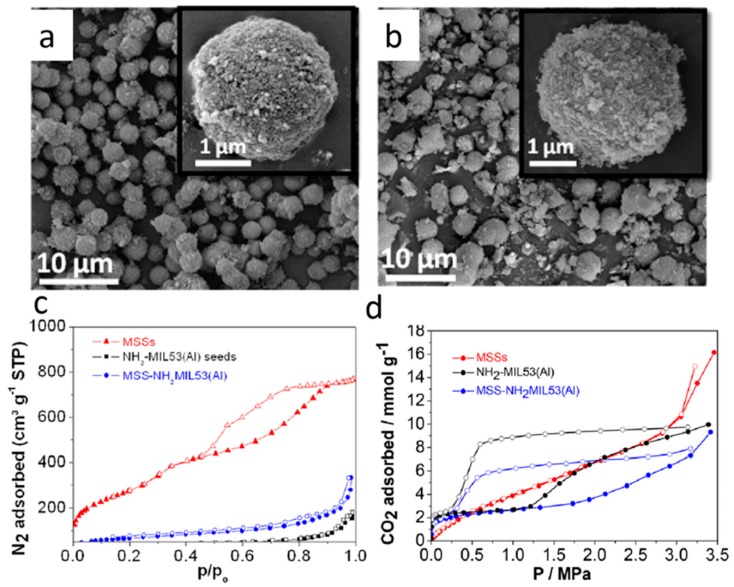
SEM images of SiO_2_ coated with NH_2_-MIL-53(Al) crystals by (**a**) an ex situ seeding process with mesoporous silica spheres (MSSs) added into NH_2_-MIL-53(Al) seeds in *N*,*N*-dimethylformamide (DMF), or by (**b**) in situ seeding with MSSs were added to the synthesis gel of NH_2_-MIL-53(Al) seeds. (**c**) Adsorption–desorption isotherms of N_2_ at −196 °C for SiO_2_, NH_2_-MIL-53(Al), seeds and SiO_2_-NH_2_MIL53(Al) samples. (**d**) Adsorption–desorption isotherms of CO_2_ at 0 °C for SiO_2_, NH_2_-MIL-53(Al), and SiO_2_-NH_2_MIL53(Al) samples. Solid and open symbols correspond to adsorption and desorption, respectively. Reproduced from [110], with permission from Elsevier, 2016.

For CO_2_ adsorption, porous/mesoporous silica materials are usually utilized as seeds, and MOF crystals are in situ synthesized on the seed to form MOF/SiO_2_ adsorbent [109]. As Sorribas et al. reported, the prepared mesoporous silica spheres (MSSs), MCM-41, reacts in situ with the precursors of Al(NO_3_)_3_·H_2_O and NH_2_-H_2_BDC to form the MOF/SiO_2_ composites through a layer-by-layer process (Figure 7) [110,111]. The adsorption amount of CO_2_ can reach 10 mmol/g when the materials are applied as a CO_2_ adsorbent due to the breathing behavior of the mesoporous silica core and microporous MOF shell. Through a hydrothermal process, MOF@SiO_2_ composites have also been reported by growing MOF nanocrystals on an SBA-15 or MCM-41 mesoporous silica matrix, which works as the structure-directing agent to guide the growth of MOFs [51,66]. As a CO_2_ adsorbent, the obtained composite material exhibits higher specific surface area than the original MOF material, and the material has a higher adsorption amount and a faster adsorption rate for CO_2_.

In other processes, MOF crystals as the core or dispersed phase are first prepared, and then silica is formed via a sol-gel process under the catalysis of acid and alkali to form MOFs/SiO_2_. Through a sol–gel process, Ulker et al. mixed the tetraethoxysilane (TEOS, SiO_2_ precursor) with the prepared HKUST-1 crystals to form an HKUST-1-doped and dispersed silica aerogel composite material [67]. Given the synergistic effect of MOFs and silica aerogel on gas adsorption, the composite has high CO_2_ adsorption capacity and can be used for carbon dioxide adsorption and the storage/separation of gas mixtures.

#### 3.2.2. Adsorption of Water Vapor

**Figure 8:**
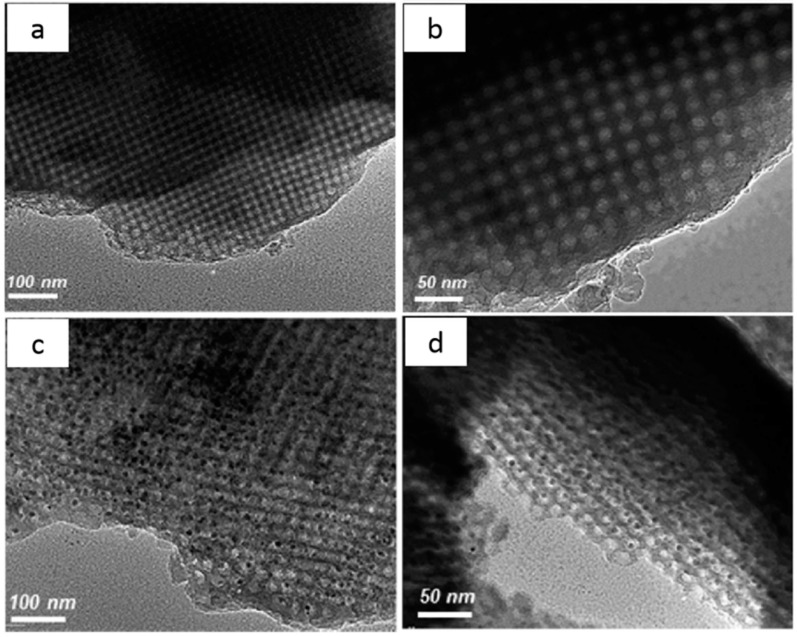
HRTEM micrographs of (**a**,**b**) NH_2_-FDU-12 silica matrix and (**c**,**d**) HKUST-1/FDU-12 composite viewed at lower (**a**,**c**) and higher (**b**,**d**) magnifications. Reproduced from [112], with permission from the Royal Society of Chemistry, 2017.

Silica can be used to enhance the water stability of MOFs, and their composites can be used to adsorb water or other moist gases. Using a microwave-assisted sol-gel process, Uma et al. fabricated an MIL-101(Cr)-SiO_2_ composite with uniform mesopores; they found that the material has high adsorption efficiency to water vapor, and the water stability of the composite is significantly enhanced by the addition of silica [68]. Mazaj et al. first prepared amino-functionalized MOFs by modifying FDU-12 with aminopropyltriethoxysilane; then, the MOFs were immersed into a Cu^2+^ solution to obtain an HKUST-1@NH_2_-FDU-12 composite material with copper ions as ligands (Figure 8) [112]. In this composite, HKUST-1 is loaded into the pores of the silica matrix, and the hydrophobic silica provides a layer of protection for the MOFs, which significantly improves the structural stability and avoids the hydrolysis of pure HKUST-1 in direct contact with water. The large specific surface area of the MOFs and the structural stability of silica effectively increase the adsorption efficiency of the composite materials, which can be used to continuously capture humid gases.

#### 3.2.3. Adsorption of Other Gases

In addition to the adsorption of carbon dioxide and water vapor, MOF/SiO_2_ composites can also be used for the adsorption and removal of sulfur-containing gases, such as H_2_S. Using an ultrasound-assisted in situ process, Saeedirad et al. grew ZIF-8 crystals on three kinds of mesoporous silica templates to form three kinds of composites, namely, MCM-41@ZIF-8, SBA-15@ZIF-8, and UVM-7@ZIF-8 [69]. The composite material performed well in the adsorption desulfurization of H_2_S and CH_3_CH_2_SH and had good recycling performance. Its absorption capacity was only reduced by approximately 10% after hydrogen sulfide and ethanethiol adsorption four times.

### 3.3. Application in the Field of Catalysis

As a kind of crystalline material containing metal or metal cluster junctions, metal–organic skeleton materials are expected to become heterogeneous catalysts or catalyst carriers [113,114,115,116,117,118,119,120]. MOF materials provide a highly tunable platform for structure and performance, integrating all the properties required for catalysis, including high surface area, high porosity, active metal sites, recyclability, and high crystallinity [55,121,122,123,124]. However, the active site of MOFs must be protected, especially in the high-temperature or high-oxidation environment of industrial catalysis, and the composite of silica/MOF material can effectively solve this problem [125]. Silica not only provides MOFs with thermal stability and structural integrity but also maintains the high availability of the active sites of MOFs.

#### 3.3.1. MOFs/SiO_2_ as Direct Catalyst

**Figure 9:**
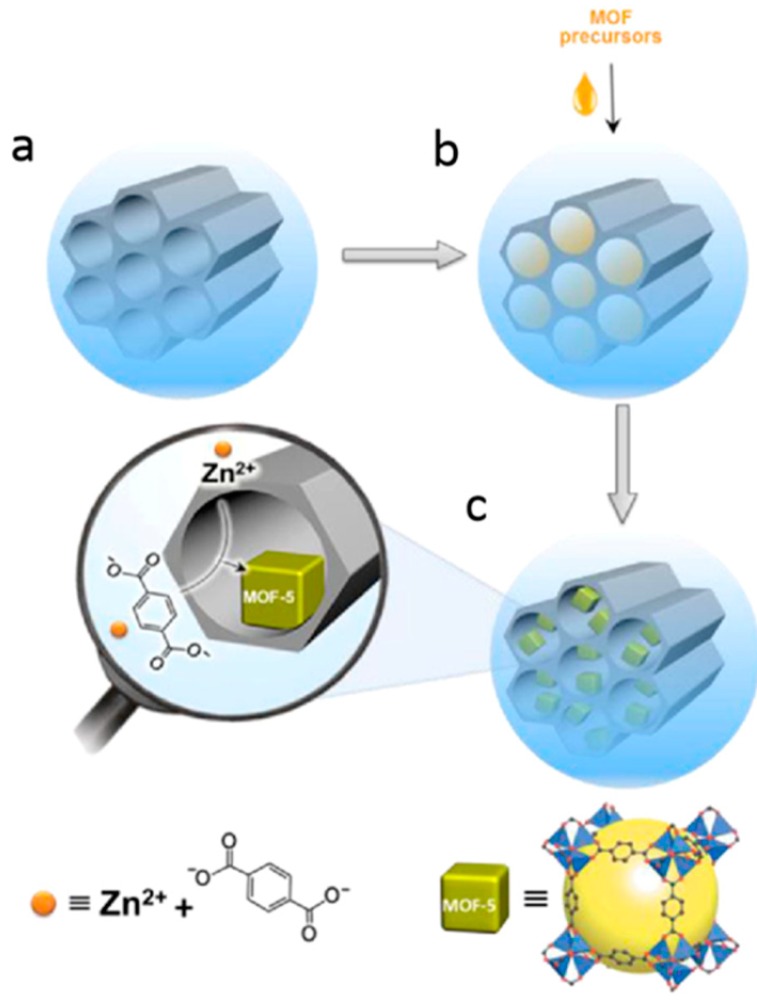
(**a**) Dispersion of mesoporous silica (SBA-15) in hydrophobic n-octane; (**b**) addition of the hydrophilic *N*,*N*-dimethylformamide (DMF) solution containing MOF precursors (metal ions and ligands); (**c**) formation of MOFs at 120 °C for 24 h. Reproduced from [70], with permission from American Chemical Society, 2018.

MOF/silica composites for catalysis can be prepared by the in situ synthesis of MOF crystals in the pores of mesoporous silica. The macroscopic morphology and crystal defects greatly affect the catalytic performance of MOF composites. Karimi et al. obtained MOF-5 crystals with different morphologies by growing MOF-5 crystals on SBA-15 mesoporous silica at different concentrations [126]. Flower-like to nanorod-like MOF-5 crystals have been controllably fabricated by regulating the concentration of silica, which could guide the directional crystallization and growth of MOFs. Among them, nanorod-shaped MOF/silica composites were found to be highly selective to the ortho-products in the catalytic Friedel–Crafts alkylation reaction, showing superior catalytic performance to pure MOF-5 materials. Kou et al. first prepared SBA-15 mesoporous silica and then grew MOF crystals in silica nanopore in a double solvent containing hydrophobic solvent and a hydrophilic solution containing MOF precursors [70]. Based on a hydrophobic n-octane solvent and hydrophilic *N*,*N*-dimethylformamide (DMF) solvent containing MOF precursor, an MOF-5@SBA-15 composite was prepared (Figure 9). Contrary to the pure MOFs, the structure of the composites remained undamaged for 8 h in a humid environment, which indicated that the water stability of MOF-5 was significantly improved by the support and protection provided by mesoporous silica. With this material as the catalyst for the Friedel–Crafts alkylation reaction, the conversion of benzyl bromide reached up to 100% within 3 h, which is considerably higher than that of pure MOF-5. In addition, Song et al. soaked bulk silica in HKUST-1 precursor solution directly and obtained HKUST-1-SiO_2_ composite materials [71]. With the composite as catalyst, the oxidations of propylbenzene, 1,2,3,4-tetrahydronaphthalene, diphenyl methane, and fluorene were carried out, and the substrates, propylbenzene, and 1,2,3,4-tetrahydronaphthalene were oxidized to the respective ketones in >90% yields and >99% selectivity. The enhancement of the catalytic performance is thought to arise partially from the support of the silica monoliths. Furthermore, this type of HKUST-1-SiO_2_ is easily recovered by simple filtration and subsequently used in the successive 12 cycles without an obvious loss in conversion (from 95% to >99%), which presents the remarkable catalytic stability of the HKUST-1–SiO_2_ composite.

Another commonly used strategy for the preparation of MOF/silica composite catalyst materials is to first synthesize MOF materials and then grow a silica shell material on it by the sol–gel method. Ying et al. first prepared MIL-101(Cr) and then wrapped a very thin layer of mesoporous silica on the surface to fabricate a hydrophobic MIL-101(Cr)@mSiO_2_ composite and utilized it to catalyze the oxidation reaction of indene with H_2_O_2_ in acetonitrile [80]. Considering the reusability of the catalyst, 82% of the initial conversion is retained for MIL-101(Cr)@mSiO_2_ after three runs, while only 46% of the initial conversion is still present for MIL-101(Cr). Mesoporous silica not only enhances the stability of MOF materials because of its hydrophobicity but also shows improved catalytic activity and recyclability. Shalygin et al. mixed MOF powders and silica sol and then obtained an aerogel composite through a gelation of silica sol, separation, and a drying process [127]. This composite aerogel material has high selectivity in the reaction of the catalytic oxidation of styrene into phenylacetaldehyde and can be used as a catalytic flow reactor for the continuous preparation of styrene and phenylacetaldehyde.

#### 3.3.2. MOF/SiO_2_ Composite as Catalyst Support

**Figure 10:**
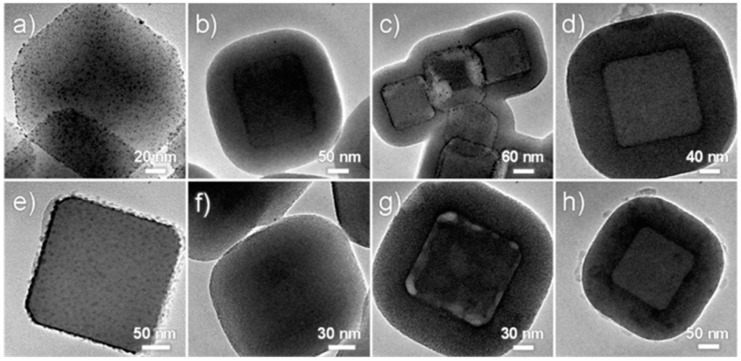
TEM images for (**a**) ZIF-8@Au after calcination, (**b**) ZIF-8@Au@mSiO_2_ before calcination, (**c**) ZIF-8@Au@mSiO_2_ after calcination, (**d**) ZIF-8@Au@mSiO_2_@ZIF-8, (**e**) ZIF-8@Cu after calcination, (**f**) ZIF-8@Cu@mSiO_2_ before calcination, (**g**) ZIF-8@Cu@mSiO_2_ after calcination, and (**h**) ZIF-8@Cu@mSiO_2_@ZIF-8. Reproduced from [72], with permission from American Chemical Society, 2014.

Maintaining the catalytic activity of active metal/metal oxide nanoparticles and improving their stability are problems that need to be solved in the field of catalysis. Encapsulating the unstable catalyst particles into MOF materials is an effective approach that can address these problems [128]. MOFs were proven to be suitable carriers for noble metal nanoparticle catalysts due to their large specific surface area and abundant pores [129]. The MOF material has uniform micropores or mesopores, which can effectively prevent particle aggregation and facilitate the transfer and diffusion of guest molecules. Moreover, MOF materials are easy to be separated and can be reused, effectively extending the service life of catalyst and reducing environmental pollution. However, MOFs have limited thermal and mechanical stability [130], which could be enhanced by silica protection layer. Li et al. established a general method to “armorize” MOF materials by coating them with silica [72]. The first step of the method is to coat a layer of gel filamentous microporous silica shell on the surface of MOFs with the help of surfactant and then grow a layer of MOF shell on the surface to prepare a composite material with an MOF@mSiO_2_@MOF core–shell structure. This structure could improve the mechanical strength, hardness, and toughness of the MOF material. The prepared ZIF-8@mSiO_2_@ZIF-8 composite has been proven to have excellent catalytic performance in the catalytic reduction of 4-nitrophenol reaction by NaBH_4_ in water media after loading metal particles such as gold/copper. Similarly, Pascanu et al. fixed Pd metal onto the pores of the MIL-88B-NH_2_ material, coated the material surface with a layer of mesoporous silica coating, and finally fabricated Pd@MIL-88B-NH_2_@nano-SiO_2_ double-supported nano-Pd catalysts [73]. The materials exhibit high catalytic activity in the oxidation of benzyl alcohol without using any bubbling device under atmospheric pressure. Under continuous flow, the composite catalyst can withstand continuous operation at 110 °C for 7 days without deactivation. During this period, no metal leaching was observed, and the material maintained its structural integrity. To enhance the mechanical properties (hardness and toughness), Li et al. reported a general synthetic approach to coat microporous MOFs and their derivatives with an enforcing shell of mesoporous silica (mSiO_2_). With ZIF-8@Au and ZIF-8@Cu utilized as binary solid cores, meosporous silica layers were wrapped on them (Figure 10) [131]. The excellent accessibility of the porous silica-wrapped MOFs and their metal-containing nanocomposites showed excellent catalytic performance using the reduction of 4-nitrophenol as a model reaction with the reaction proceeded rapidly with a conversion over 99% in ca. 14 min.

#### 3.3.3. MOF/SiO_2_ Composite-Derived Metal/Silica Catalyst 

Using a ligand calcining or dissolving process, MOF/silica composites can directly derivatize metal/silica composites with good dispersibility and good catalytic activity [132,133]. Desai et al. loaded cobalt or cobalt–aluminum complexes onto the zirconium nodes of a zirconium-based MOF material NU-1000 using the osmotic method and then grew SiO_2_ in the voids of MOFs [134]. Finally, after removing the double organic ligands, the Co_2_-Zr_6_@SiO_2_ and AlCoZr_6_@SiO_2_ polymetallic site oxide cluster composites protected by silica carrier were prepared. The composite material was stable and firm, and its cobalt site exhibited high catalytic activity in the oxidation of benzyl alcohol to benzaldehyde. By calcining a ZIF-67/mesoporous silica composite, Zhou et al. prepared silica–Co/N carbon nanotube (CNT) composites [135]. The SiO_2_ shell not only prevented the rapid accumulation of Co nanoparticles in ZIF-67, but also provided a unique external “sieve” to induce the catalytic growth of CNTs. The prepared carbon nanotubes had a good 3D framework structure, good stability, and good solvent tolerance, and the composite exhibited excellent electrocatalytic performance, even exceeding some commercial catalysts.

### 3.4. Application in Biomedicine

Metal–organic matrix composites have attracted considerable attention in the fields of separation, adsorption, and catalysis; however, their application in biomedicine remains a challenge [136,137]. MOF composites have the advantages of flexible composition, adjustable pore size, and high crystallinity, and they have attracted considerable attention in biomedical fields, especially in drug delivery, angiography, diagnosis, and treatment [138,139,140]. The composite of silica shell and MOFs can provide several advantages, including enhancing the water dispersion and biocompatibility of the materials, and the condensation of organosilica precursors can further functionalize the MOF materials [141,142]. Rieter et al. first synthesized MOF nanorods with lanthanide metal ions and terephthalic acid; they then modified the nanorods with polyvinylpyrrolidone (PVP) and coated them with silica materials through a sol–gel process [143]. The obtained MOF/SiO_2_ composite materials showed good performance in drug sustained release and biomarkers.

**Figure 11:**
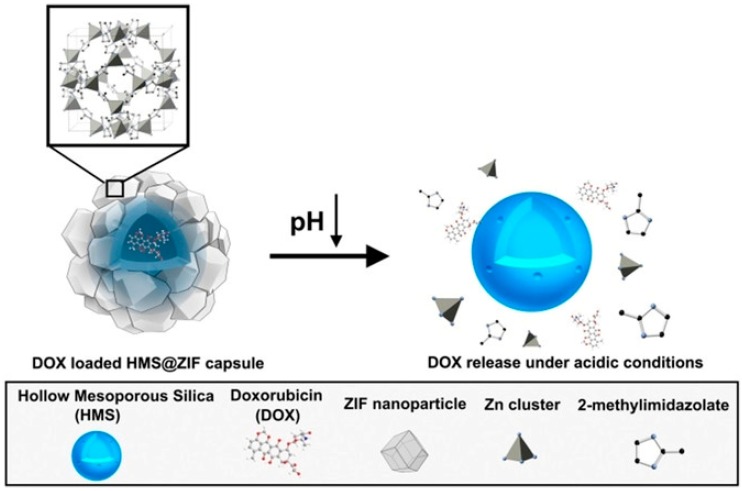
Synthesis of hollow mesoporous silica (HMS)/MOFs with encapsulated anticancer drug molecules. Reproduced from [74], with permission from Wiley-VCH, 2018.

For drug release, to avoid the rapid and uncontrolled release of the original drug, stimulus-responsive MOF composite materials have attracted interest. Zeolite imidazole MOFs, such as ZIF-8, synthesized from dimethylimidazole and zinc ions, have pH stimulation responsiveness. They are very stable under physiological conditions but dissociate under acidic conditions. Hence, the material is highly suitable for drug loading and targeted release. Jia et al. loaded the anticancer drug DOX into hollow mesoporous silica (HMS) and then wrapped ZIF-8 to obtain a DOX/HMS@ZIF-8 composite capsule (Figure 11) [74]. DOX in the HMS@ZIF-8 composite was not released under physiological conditions (pH 7.4); it was only released at low pH (4–6). HMS@ZIF-8 has excellent cell compatibility and can be used to assemble pH-responsive drug delivery systems. Zou et al. also fabricated a mesoporous layer on ZIF-8 particles with different morphologies and removed the ZIF-8 to obtain the HMS, which is suitable for drug delivery due to their excellent biocompatibility, large cavity, and controllable morphology [75].

The advantage of MOF/silica composites for biomedicine is that they combine the synergy of inorganic and organic chemistry, have high porosity and rich organic functional groups, and have biocompatibility and good stability. With carboxyl-functionalized silica beads as a matrix, Liu et al. prepared a SiO_2_@EuTTA composite by coating a layer of lanthanum with thiophene trifluoroacetone on the silica. After grafting a ZIF-8 shell on the composite, they prepared a SiO_2_@EuTTA@ZIF-8 core–shell composite [144]. The composite showed high selectivity and sensitivity to detect Cu^2+^ in environmental or biological solution systems. 

### 3.5. Other Applications

**Figure 12:**
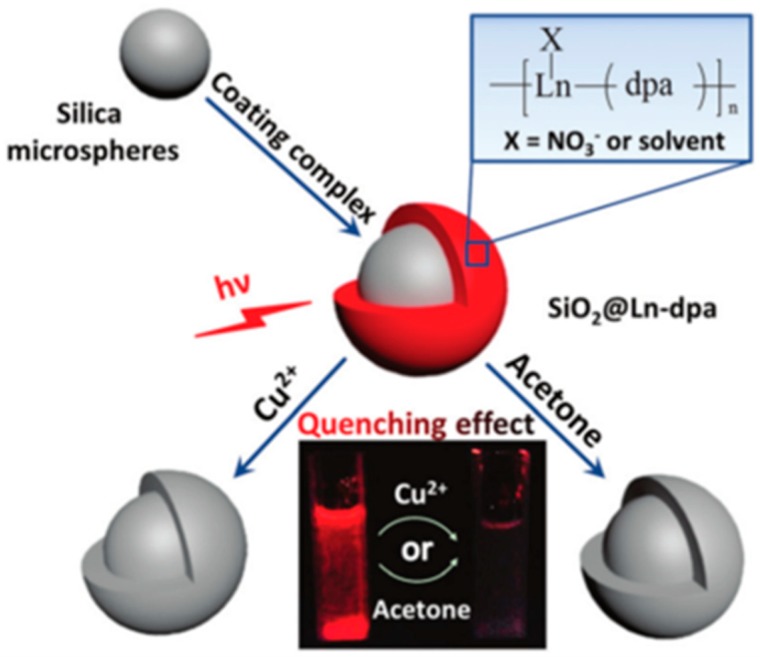
Schematic of synthesis and sensing process of SiO_2_@Ln-dpa core–shell microspheres. Reproduced from [77], with permission from the Royal Society of Chemistry, 2016.

MOFs/SiO_2_ have shown versatile applications by combining the high specific surface area, high porosity, and adjustable structure of MOFs and the high stability and functionality of SiO_2_. In addition to the above applications, the composite material could also be utilized as an electrode material. Its void structure can accommodate huge volume changes and maintain the structural integrity and long cycle stability of the electrode. Sun et al. synthesized a copper-based MOF material (Cu-MOF), which was coated with a layer of silica and finally carbonized at 700 °C to obtain a silica–copper–carbon nanocomposite electrode material [145]. For gas separation, porous silica provides a growing environment and support for MOF films, providing a new method for the preparation of gas-separated MOF films. Using a layer-by-layer process, Shekhah et al. fabricated MOF films with mesoporous silica foam as a template to form highly crystalline HKUST-1 and ZIF-8, which showed high performance for gas separation [76]. Lanthanide MOF materials have great potential in the fields of luminescent dopants, solid-state lighting, integrated optics, optical communication, and solar cells [146]. In the lanthanide MOF/SiO_2_ composite, lanthanide ions are connected to the Si–O network by covalent bonds, and the silica network provides uniform dispersion and luminescence stability for the lanthanide metal. Using a hydrothermal process, Lian et al. prepared SiO_2_@MOF composites (Figure 12) by coating lanthanide MOFs on carboxyl-modified silica spheres [77]. The obtained SiO_2_@MOF microspheres are suitable for a reliable sensing process for acetone and Cu^2+^. Moreover, the materials show excellent luminescent properties and have a potential applications in the development of white-light devices. 

For water treatment, the addition of silica can improve the dispersion of MOFs in composite materials, which is extremely beneficial for the adsorption of pollutants in water and has great potential in the prevention and control of water pollution [147]. Han et al. synthesized a series of SiO_2_@Al–MOF(MIL-68) composites with different silica contents, which showed ultrafast aniline adsorption and high recyclability [78]. Given the good physical and chemical properties, MOF/SiO_2_ composites could be utilized in more applications.

## 4. Conclusions and Outlook

MOFs assembled by metal ions and organic ligands are a kind of newly crystallized porous material with ultrahigh porosity and very large specific surface area, and the addition of silica further improves the stability and availability of MOF materials. In Table 1, we summarized the synthesis and application of MOF/silica composite materials in chromatographic column separation, gas adsorption, catalysis, biomedicine, and other fields. A large number of readings in the literature have shown that MOF/silica materials show excellent properties that are rarely seen in other materials. By compounding with SiO_2_ material, the stability and mechanical properties of MOF material can not only be improved, but also the matrix structure of MOFs can be optimized by different SiO_2_ structures. In addition, the specific surface area and contact ability of the material can be improved. 

The existing challenge lies in the structural control and the fabrication of composite materials with new structure. With new MOFs and their derivatives, the interface of MOFs and silica materials and the control of their morphology, including pores, will become prominent problems. A new preparation method for general MOF/SiO_2_ composites must be developed urgently. The key issues for future research are the regulation of the structure and morphology of MOFs/SiO_2_ materials and the surface modification and functionalization to obtain composites with stability, biocompatibility, and specific targeted functionality. MOF/silicon dioxide composites with high stability and structural controllability will have great application potential in the fields of environmental protection, biomedicine, and new energy.

## Figures and Tables

**Table 1:** Synthesis strategy and advanced applications of MOF/SiO_2_ nanocomposites. MOF: metal organic framework.

MOF/SiO_2_	Synthesis Strategy	Application	Ref.
UiO-66@SiO_2_	Solvothermal process to coat UiO-66 on silica core	Stationary phase for HPLC	[61]
HKUST-1-SiO_2_	Synthesis of MOFs in the mesoporous silica pores	Stationary phase for HPLC	[62]
UiO-66-NH_2_@SiO_2_	One pot synthesis of UiO-66-NH_2_ and silica gel	Stationary phase for HPLC	[63]
HKUST-1-SiO_2_	MOFs were incorporated in situ into mesoporous silica pores	Stationary phase for HPLC	[64]
Cu(BDC)-SiO_2_	MOFs nanocrystals grown in the pores of mesoporous silica	CO_2_ adsorption	[65]
MIL-101(Cr)-SiO_2_	In situ hydrothermal method	CO_2_ adsorption	[66]
HKUST-1-SiO_2_	Sol–gel method	CO_2_ adsorption	[67]
MIL-101(Cr)-SiO_2_	Microwave-assisted hydrothermal	Water vapor adsorption	[68]
ZIF-8@SiO_2_	Ultrasound-assisted in situ process	H_2_S adsorption	[69]
MOF-5@SiO_2_	Double-solvent strategy to grow MOFs inside silica pores	Catalyst	[70]
HKUST-1-SiO_2_	In situ synthesis of MOFs in porous silica monoliths	Catalyst	[71]
ZIF-8@ SiO_2_ ZIF-7@ SiO_2_ UiO-66@ SiO_2_ HKUST-1@ SiO_2_	Sol-gel process to coat silica on MOFs	Catalyst support	[72]
MIL-88B-NH_2_@ SiO_2_	Sol-gel process to coat silica on MOFs	Catalyst support	[73]
ZIF-8@ SiO_2_	Drug DOX loaded into hollow mesoporous silica and then wrapped ZIF-8	Drug Delivery	[74]
SiO_2_@ZIF-8	Mesoporous silica layer on ZIF-8 particles	Drug Delivery	[75]
HKUST-1-SiO_2_ ZIF-8-SiO_2_	Layer-by-layer grown of MOFs on silica foam	Gas separation	[76]
SiO_2_@Eu-dpa	Solvothermal process to grow MOFs on silica spheres	Fluorescence sensing	[77]
SiO_2_@MIL-68	MIL-68(Al) grow and nucleate on the surface of silica nanoparticles	Pollute removal	[78]
